# Exertional Hyponatremia Among Active Component Members of the U.S. Armed Forces, 2009–2024

**Published:** 2025-06-20

## Abstract

Exertional hyponatremia, or hyponatremia associated with exercise, occurs within 24 hours after physical activity due to a serum, plasma, or blood sodium concentration ([Na+]) below the normal reference range of 135 mEq/L. Hyponatremia can be fatal if not detected early and managed properly. From 2009 to 2024, 1,829 cases of exertional hyponatremia were diagnosed among U.S. active component service members (ACSMs), with an overall incidence rate of 8.4 cases per 100,000 person-years (p-yrs). In 2024, 134 cases of exertional hyponatremia were diagnosed among ACSMs, resulting in a crude incidence rate of 10.4 per 100,000 p-yrs. Female service members, those older than 40 years, non-Hispanic White service members, Marine Corps members, recruits, those in health care occupations, and ACSMs stationed in the Midwest U.S. had higher incidence rates of diagnosis for exertional hyponatremia than their respective counterparts. From 2009 to 2024, annual rates of incident exertional hyponatremia diagnoses peaked in 2010 (12.8 per 100,000 p-yrs) and then decreased to a low of 5.3 cases per 100,000 p-yrs in 2013. The incidence rate has fluctuated since then, rising from 6.1 per 100,000 p-yrs in 2017 to the second-highest level (11.2 per 100,000 p-yrs) in 2023 before decreasing to 10.4 per 100,000 p-yrs in 2024. Service members and their supervisors must be aware of the dangers of excessive consumption of water and the prescribed limits of water intake during prolonged physical activity, including field training exercises, personal fitness training, as well as recreational activities, particularly in hot, humid weather.

Exertional hyponatremia is a relatively rare condition, but it can be fatal if not detected early and managed properly. Exertional hyponatremia is caused by increased intake of hypotonic fluids, such as water or sports drinks, before or during strenuous physical activity, including prolonged military field training and combat operations. Exertional hyponatremia can also be caused by inappropriate secretion of a non-osmotic antidiuretic hormone due to physical exertion, resulting in increased total body and free water retention. ^1^

Exercising in hot weather continues to cause preventable injuries and deaths in young, healthy people. ^2^ Hyponatremia is particularly problematic in the military, where it can be mistaken for heat exhaustion or heat stroke. Active component military personnel are particularly susceptible to fluid and electrolyte imbalances due to intense physical exertion and demanding activities, often in hot, remote, or austere environments. ^2 , 3^

Normal plasma sodium (Na+) concentration falls between 135 and 145 milliequivalents per liter (mEq / L), which is closely regulated, along with osmolarity, to maintain proper cell size and function. ^4^ Excessive intake of sodium stimulates thirst to increase body water to maintain serum [Na ^+^ ]. ^5 , 6^ When a serum or plasma sodium concentration is less than 135 mEq / L within 24 hours after prolonged physical activity, hyponatremia or exercise-related hyponatremia occur. ^7^ There is growing evidence that hyponatremia is associated with increased morbidity, mortality, and health costs in various clinical settings and diseases. ^8 , 9^

**What are the new findings?**

Incidence rates of exertional hyponatremia changed from 2023 to 2024, with the overall incidence rate decreasing from 11.2 to 10.4 per 100,000 p-yrs. Rates increased, however, in the 25-29 years age group and in the Coast Guard, while decreasing sharply among non-Hispanic Black individuals and recruits. The highest incidence rates were observed in non-Hispanic White individuals and health care personnel.

**What is the impact on readiness and force health protection?**

Incidence rates of exertional hyponatremia among U.S. military members have fluctuated but, in general, increased in the past decade, posing a substantial health risk to U.S. military members. Exertional hyponatremia can be fatal if not recognized promptly and treated appropriately. Military members, leaders, and trainers must be vigilant for early signs of hyponatremia and intervene immediately and appropriately, while adhering to guidelines for proper hydration during physical exertion, especially during warm weather.

The incidence of hyponatremia due to a variety of activities, including endurance competitions, hiking, police training, American football, fraternity hazing, and military exercises, varies widely depending on activity duration, heat or cold stress, water availability, and consumption, and other individual risk factors. ^10^ Other important risk factors besides excessive fluid intake include exercise duration of greater than 4 hours, inadequate training for an exertional event, and high or low body mass index. ^10^ Symptoms depend on the extent and rate of decrease in serum sodium compared to baseline levels.

The fundamental characteristics of military operations, such as long-term military training and combat operations in extreme environmental conditions, mean that exertional hyponatremia continues to pose a health risk to U.S. military personnel, with the potential for significantly reducing performance and combat effectiveness. This report summarizes the frequency, rates, trends, demographic, geographic location, and military characteristics of exertional hyponatremia cases among active component service members (ACSMs) from 2009 to 2024.

## Methods

The surveillance population for this report consisted of all ACSMs of the U.S. Army, Navy, Air Force, Marine Corps, Space Force, and Coast Guard who served at any time during the surveillance period, from January 1, 2009 to December 31, 2024. All data used to determine incident exertional hyponatremia diagnoses were derived from records routinely collected and maintained in the Defense Medical Surveillance System (DMSS). Those records document both ambulatory encounters and hospitalizations of ACSMs of the U.S. Armed Forces in fixed military and civilian (if reimbursed through the Military Health System) hospitals and clinics worldwide.

A case of exertional hyponatremia was defined as 1) a hospitalization or ambulatory visit with a primary (first-listed) diagnosis of “hypo-osmolality and/or hyponatremia” (International Classification of Diseases, 9th and 10th Revisions, ICD-9: 276.1, ICD-10: E87.1) and no other illness or injury-specific diagnoses (ICD-9: 001 – 999, ICD-10: ‘A’ – ‘U’) in any diagnostic position or 2) both a diagnosis of “hypo-osmolality and / or hyponatremia” (ICD-9: 276.1, ICD-10: E87.1) and at least 1 of the following within the first 3 diagnostic positions (dx1 – dx3): “fluid overload” (ICD-9: 276.9; ICD-10: E87.70, E87.79), “alteration of consciousness” (ICD-9: 780.0*, ICD-10: R40.*), “convulsions” (ICD-9: 780.39, ICD-10: R56.9), “altered mental status” (ICD-9: 780.97, ICD-10: R41.82), “effects of heat / light” (ICD-9: 992.0–992.9, ICD-10: T67.0* – T67.9*), or “rhabdomyolysis” (ICD-9: 728.88, ICD-10: M62.82). ^11^

Medical encounters were excluded from case-defining events if the associated records listed diagnoses in any diagnostic position that included alcohol or illicit drug abuse; psychosis, depression, or other major mental disorders; endocrine disorders; kidney diseases; intestinal infectious diseases; cancers; major traumatic injuries; or complications of medical care. An individual could be considered a case of exertional hyponatremia only once per calendar year. Incidence rates were calculated as cases of hyponatremia per 100,000 person-years (p-yrs) of active component service.

For health surveillance purposes, recruits were identified as active component members assigned to service-specific training locations during coincident service-specific basic training periods. Recruits were considered as a separate category of enlisted service members in summaries of exertional hyponatremia by military grade overall.

## Results

In 2024, 134 cases of exertional hyponatremia were diagnosed among ACSMs, resulting in a crude incidence rate of 10.4 per 100,000 p-yrs, a decrease from 11.2 per 100,000 p-yrs in 2023. From 2009 to 2024, there were 1,829 incident diagnoses of exertional hyponatremia among ACSMs resulting in a crude overall incidence rate of 8.4 cases per 100,000 p-yrs. **Table 1** presents the incident cases and rates of exertional hyponatremia according to demographic characteristics.

**TABLE 1.** Incident Cases ^a^ and Rates ^b^ of Exertional Hyponatremia, Active Component, U.S. Armed Forces, January 2009–December 2024

	2024		Total 2009–2024	
	No.	Rate ^b^	No.	Rate ^b^
Total	134	10.4	1,829	8.4
Sex				
Male	107	10.1	1,534	8.3
Female	27	11.7	295	8.5
Age Group, y				
<20	12	14.6	223	16.0
20–24	30	7.7	491	7.1
25–29	36	11.8	363	7.0
30–34	12	5.6	236	6.7
35–39	14	8.4	215	8.3
40+	30	22.0	301	13.2
Race and ethnicity				
White, non-Hispanic	78	11.7	1,151	9.1
Black, non-Hispanic	20	9.5	246	7.2
Hispanic	19	7.4	226	6.8
Other / unknown	17	10.6	206	8.1
Service branch				
Army	55	12.5	652	8.3
Navy	21	6.5	288	5.5
Air Force	29	9.0	369	7.2
Marine Corps	24	14.2	470	15.8
Coast Guard	5	12.6	50	7.7
Military rank				
Enlisted	81	7.9	1,130	6.4
Officer	40	16.5	417	10.8
Recruit	11	47.3	280	64.0
Military occupation				
Combat-specific ^c^	18	11.1	309	10.2
Motor transport	3	7.1	39	5.5
Pilot / air crew	5	11.1	49	6.3
Repair / engineering	21	5.8	335	5.2
Communications / intelligence	32	11.5	327	6.9
Health care	13	12.3	137	7.4
Other	42	13.9	633	14.3
Home of record				
Midwest	21	10.7	327	8.5
Northeast	15	9.7	264	9.6
South	57	10.1	779	8.4
West	31	10.3	363	7.3
Other / unknown	10	12.7	96	9.5

Abbreviations: No., number; y, years.

a One case per person per year.

b Rate per 100,000 person-years.

c Infantry / artillery / combat engineering / armor.

In 2024, female ACSMs had a higher annual incidence rate (11.7 per 100,000 p-yrs) than males (10.1 per 100,000 p-yrs), although both sexes showed a decrease. Service members aged 40 years and older showed the highest incidence rate, followed by those younger than 20 years (22.0 and 14.6 per 100,000 p-yrs, respectively). It is notable that the incidence of those aged 25-29 years increased noticeably in 2024 compared to 2023 (11.8 and 7.8 per 100,000 p-yrs, respectively). Another remarkable change was in relation to racial and ethnic groups: non-Hispanic White service members had the highest incidence rate (11.7 per 100,000 p-yrs) compared to other racial and ethnic groups, especially non-Hispanic Black service members, who previously demonstrated the highest incidence rate. As with overall 2009-2024 rates, Marine Corps members had the highest incidence rate in 2024 (14.2 per 100,000 p-yrs) compared to other services. Meanwhile, the incidence rate in the Coast Guard increased greatly, from 2.6 per 100,000 p-yrs in 2023 to 12.6 per 100,000 p-yrs in 2024.

There were 11 cases of exertional hyponatremia among recruits in 2024, an approximately 50% decrease from 2023 (47.3 to 90.2 per 100,000 p-yrs). Service members in health care occupations had the highest incidence rate in 2024, an approximately 18% increase from 2023 (12.3 to 10.4 per 100,000 p-yrs), excluding the ‘other’ or unknown occupation group.

**FIGURE 1.**
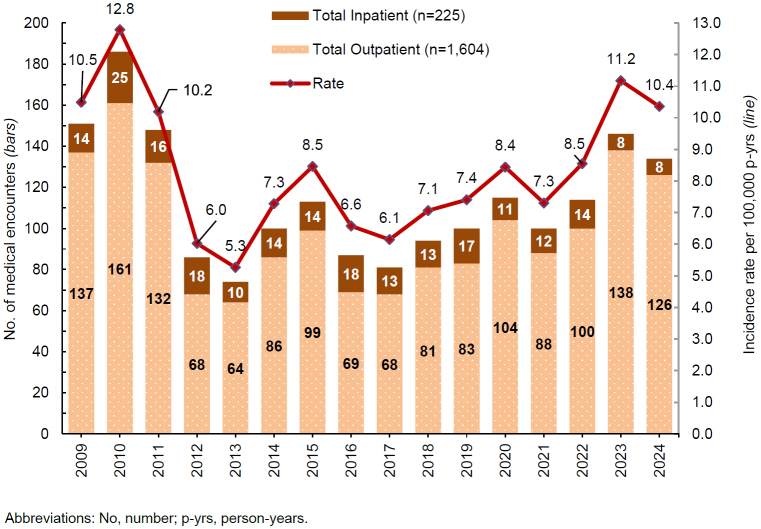
Annual Incident Cases and Rates of Exertional Hyponatremia, Active Component, U.S. Armed Forces, 2009–2024

**FIGURE 2.**
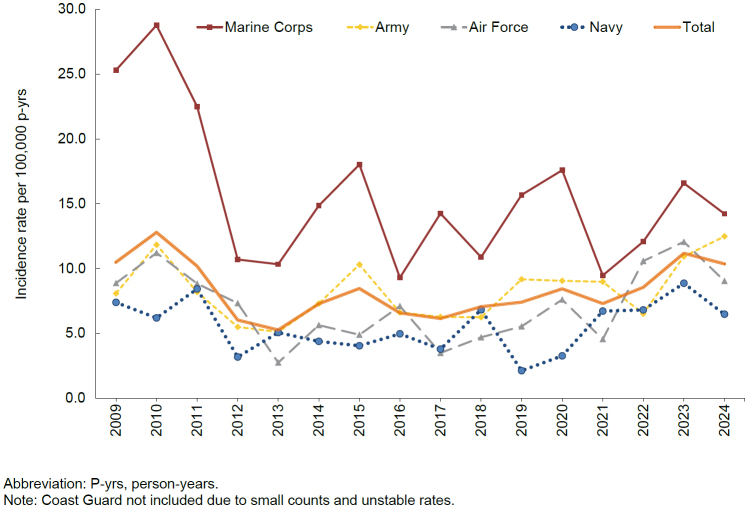
Annual Incidence Rates of Exertional Hyponatremia by Service, Active Component, U.S. Armed Forces, 2009–2024

**FIGURE 3.**
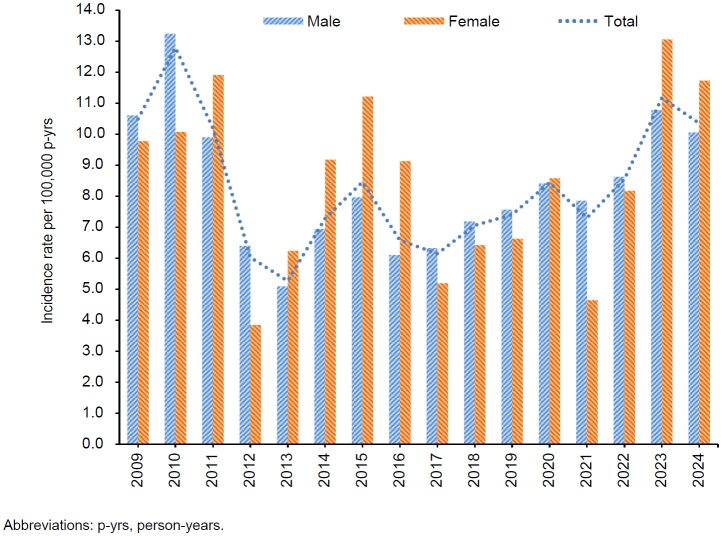
Annual Incident Rates of Exertional Hyponatremia by Sex, Active Component, U.S. Armed Forces, 2009–2024

**Figure 1** presents annual incident cases and rates of exertional hyponatremia among ACSMs. Between 2009 and 2024, the crude annual rates of incident exertional hyponatremia diagnoses peaked in 2010 (12.8 per 100,000 p-yrs) and then decreased to the lowest level, 5.3 cases per 100,000 p-yrs, in 2013. During the ensuring decade, rates fluctuated but generally trended upward, rising from a low of 6.1 cases per 100,000 p-yrs in 2017 to a peak of 11.2 cases per 100,000 p-yrs in 2023, before decreasing to 10.4 per 100,000 p-yrs in 2024. The annual incidence of exertional hyponatremia diagnosis was significantly higher in the Marine Corps than in any other service branch **(Figure 2)** . The incidence of exertional hyponatremia fluctuated more among women than men **(Figure 3)** . During the 16-year surveillance period, 87.7% (n=1,604) of all cases were diagnosed and treated without hospitalization (data not shown).

During the surveillance period, exertional hyponatremia cases were diagnosed at more than 150 U.S. military installations and geographic locations worldwide, but 17 U.S. installations contributed 20 or more cases each and accounted for 49.9% of the total cases **(Table 2)** . Marine Corps Recruit Depot (MCRD) Parris Island, SC, reported 195 cases of exertional hyponatremia, the highest in the DOD.

**TABLE 2.** Incident Cases of Exertional Hyponatremia by Installation with at Least 20 Cases During the Period, Active Component, U.S. Armed Forces, 2009–2024

Location of Diagnosis	No.	% Total
MCRD Parris Island / Beaufort, SC	195	10.7
Fort Benning, GA	149	8.1
JBSA-Lackland AFB, TX	63	3.4
MCB Camp Lejeune / Cherry Point, NC	58	3.2
Fort Bragg, NC	56	3.1
NMC San Diego, CA	52	2.8
MCB Camp Pendleton, CA	44	2.4
NMC Portsmouth, VA	44	2.4
Walter Reed NMMC, MD	44	2.4
Fort Cavazos, TX	32	1.7
Fort Campbell, KY	30	1.6
Fort Schafter, HI	28	1.5
MCB Quantico, VA	26	1.4
Fort Belvoir, VA	25	1.4
Fort Carson, CO	25	1.4
Fort Jackson, SC	21	1.1
NH Jacksonville, FL	21	1.1
Other / unknown locations	916	50.1
Total	1,829	100

Abbreviations: No., number; MCRD, Marine Corps Recruit Depot; JBSA, Joint Base San Antonio; AFB, Air Force Base; MCB, Marine Corps Base; NMC, Naval Medical Center; NMMC, National Military Medical Center; NH, Naval Hospital. Note: Recruit training locations include Fort Jackson, Fort Benning, Fort Sill, Fort Leonard Wood, Lackland Air Force Base, Keesler Air Force Base, Coast Guard Training Center Cape May, MCRD Parris Island, MCRD San Diego, and Naval Station Great Lakes. Referral centers include Walter Reed NMMC, NMC San Diego, and NMC Portsmouth.

## Discussion

Incidence rates of exertional hyponatremia fluctuated over the past decade, increasing from 6.1 per 100,000 p-yrs in 2017 to 11.2 per 100,000 p-yrs in 2023, before decreasing slightly to 10.4 per 100,000 p-yrs in 2024. Notable changes in incidence rates of exertional hyponatremia were observed among several demographic groups in 2024 compared to 2023. Although reports on the association between sex and hyponatremia present conflicting results, ^12 , 13^ many studies report that sex is not a significant risk factor for hyponatremia. ^16 - 17^ Further investigation and ongoing monitoring may be warranted, however, to effectively prevent exertional hyponatremia, especially in women, given the greater variability in incidence in women than in men.

The age group with the highest incidence rate in 2024 was the 40 years and older age group, which had decreased considerably from 2023. According to the literature, increasing age is a strong independent risk factor for both hyponatremia and hypernatremia. ^14^ Unlike other age groups that showed declines compared to 2023, the incidence rate in 2024 increased remarkably in the 25-29-year age group. Investigation of the reasons for this change, to identify modifiable risk factors that can be targeted in prevention programs, is warranted.

Since 1999, differences in incidence rates between non-Hispanic White and non-Hispanic Black service members have ranged from large to small or insignificant differences. ^10 , 18^ During the 16-year surveillance period, incidence rates among non-Hispanic White service members were higher than those among non-Hispanic Black service members, in all but 6 years: 2005, 2016, 2018, 2019, 2022, 2023. Several studies have reported conflicting results about the potential association between hyponatremia and race or ethnicity. Some studies have reported a higher prevalence in African Americans and non-Hispanic Black individuals, ^19^ whereas others have reported a trend of lower proportions of African American hospital admissions with hyponatremia. ^15^

Unlike other service branches, 3 of which showed a decline compared to 2023, the Coast Guard showed a noticeable increase in the incidence rate of its members in 2024. There was also significant variation in incidence rates across military grade (i.e., rank), with recruits consistently having the highest incidence rates.

Several important limitations should be considered when interpreting the results of this analysis. First, there is no diagnostic code specific for exertional hyponatremia. This lack of specificity may result in inclusion of some non-exertional cases of hyponatremia, thus overestimating the true rate. Consequently, the results of this analysis should be considered estimates of the actual incidence of symptomatic exertional hyponatremia from excessive water consumption among U.S. military members.

In addition, the accuracy of estimated numbers, rates, trends, and correlates of risk depends on the completeness and accuracy of diagnoses that are documented in standardized records of relevant medical encounters. Nonetheless, the decline in the number of diagnoses presenting with exertional hyponatremia may reflect increased awareness, concern, and aggressive management of early cases by military supervisors and primary health care providers.

Finally, recruits were identified using an algorithm based on age, rank, location, and time in service, which was only an approximation and likely resulted in some misclassification of recruit training status.

Given the remarkable changes from 2023 to 2024 in the incidence rate of exertional hyponatremia for numerous demographic characteristics analyzed, continued emphasis should be placed on how to effectively manage the condition, including prevention, identification, and treatment methods through close monitoring. Hyponatremia is treated primarily by managing the underlying cause (i.e., heart failure) and free water restriction, ^20^ focusing on pre-hospital care through rapid on-site emergency medical service assessment and hospital management in emergency and inpatient settings. ^21^ Depending on the physical demands of military operations and prevailing environmental conditions, replacement fluid composition may vary. ^22^

Exertional hyponatremia must be differentiated from heat illness to avoid inappropriate treatment and adverse outcomes and, instead, accurately diagnose and appropriately treat the condition based on observed signs and symptoms. Appropriately trained personnel should be able to recognize the signs of possible hyponatremia, such as excessive fluid intake, changes in mental status, vomiting, poor eating habits, abdominal bloating, and large amounts of clear urine. ^2 , 23 , 24^

Due to the variety of underlying causes, individualized management based on each service member's overall health may be the best approach to prevent exertional hyponatremia. Effective and collaborative management consistent with current policy and guidance for commanders is crucial for prevention of exertional hyponatremia **(Table 3)** . ^23^ To reduce risk of exertional hyponatremia, service members of all ranks should be cognizant of mitigation measures such as fluid and electrolyte replacement guidelines, identification of high-risk individuals, and the importance vigilance during associated activities. ^23^

**TABLE 3.** TRADOC Recommendations ^a^ for Continuous Work Duration and Fluid Replacement in Warm and Hot Environments

		Easy Work		Moderate Work		Heavy Work		Very Heavy Work	
Heat Category	WBGT Index (°F)	Work (min)	Water Intake (qt / hr)	Work (min)	Water Intake (qt / hr)	Work (min)	Water Intake (qt / hr)	Work (min)	Water Intake (qt / hr)
1 (white)	78 – 81.9	NL ^b^	½	NL ^b^	¾	110	¾	45	¾
2 (green)	82 – 84.9	NL ^b^	½	NL ^b^	1	70	1	40	1
3 (yellow)	85 – 87.9	NL ^b^	¾	NL ^b^	1	60	1	25	1
4 (red)	88 – 89.9	NL ^b^	¾	180	1¼	50	1¼	20	1¼
5 (black)	>90	NL ^b^	1	70	1½	45	1½	20	1½

Notes:
Applies to average-sized and heat-acclimatized service member wearing the operational camouflage pattern uniform. Fluid needs can vary based on individual differences (± ¼ qt/hr) and exposure to full sun or shade (± ¼ qt/hr). CAUTION: Hourly fluid intake should not exceed 1½ qts. CAUTION: Daily fluid intake should not exceed 12 qts.

Abbreviations: TRADOC, Training and Doctrine Command; WBGT, wet bulb global temperature; F, Fahrenheit; min, minimum; qt, quart; hr, hour; NL, no limit.

a Reference 23 , page 24.

b No work limit per hour, up to 4 continuous hours.
